# Assessment of the Selected Health Factors by Polonia in the Greater Toronto Area in the Relation to Their Quality and Standard of Living

**DOI:** 10.3390/ijerph18031296

**Published:** 2021-02-01

**Authors:** Kamila Ziółkowska-Weiss

**Affiliations:** Department of Tourism and Regional Studies, Institute of Geography, Pedagogical University of Cracow, 30-084 Kraków, Poland; kamilazw@up.krakow.pl

**Keywords:** health, Polonia, standard of living, Toronto, quality of life

## Abstract

Quality of life constitutes an indicator of well-being, satisfaction or happiness resulting from one’s existence. It is often referred to as a standard of living. In general, it is contentment with the fulfilment of one’s needs. The main objective of the article is to describe the selected components of the living standards and quality of life within the Polish community of the Greater Toronto Area which includes four regions: Halton, Peel, York and Durham. The model of mutually affecting objective factors (standard of living) and subjective factors (quality of life) will be presented. The specific factors (demographic, social, cultural, economic, legal, educational, geographical and health-related) included in field studies among the respondents and based on assigned indices influencing the quality of life in the Polish community of the Greater Toronto Area, will be demonstrated. The major goal of the paper is to present an assessment of aspects related to health factors, both in terms of objective factors (standard of living) and subjective ones (quality of life) by Polonia living in the Greater Toronto Area. Results will be shown on the basis of the survey questionnaire completed by 583 respondents. The questions focused on, among other issues, access to the healthcare system, competence of medical staff and access to sports facilities. Respondents also evaluated their satisfaction with their general health, both physical and mental, as well as the possibility of practicing sports associated with healthy lifestyle.

## 1. Introduction

Quality of life constitutes an ambiguous concept which is described very differently depending on the profession and specialty of the person who defines it (a philosopher, a poet, a physician, a priest, etc.) [1]. Most researchers dealing with this issue state that there is no unambiguous, precisely defined definition of this notion. According to Delkey and Rurke, quality of life is the sensation of prosperity, satisfaction or dissatisfaction with life, i.e., happiness or unhappiness [2].

In medicine, the concept of quality of life did not appear until the 1970s. Earlier, it had been specified by a definition of health formulated by the World Health Organization:

*“Quality of life is defined as individuals’ perception of their position in life in the context of the culture and value systems in which they live and in relation to their goals, expectations, standards and concerns. It is a broad ranging concept affected in a complex way by the person’s physical health, psychological state, level of independence, social relationships, personal beliefs and their relationship to salient features of their environment”.*

[3]

Human health behaviors are already being shaped in early childhood, in the process of socialization. The factors influencing formation of a particular behaviour include, among others, personal patterns at home, kindergarten, school, local community, mass media, advertising, etc. Childhood and youth constitute the decisive periods for health behaviors and lifestyles in adulthood. Health behaviors are the manners of conduct of an individual that have an impact on health, its maintenance and strengthening. It should be noted that health behaviors are not permanent patterns. They are developed throughout life under the influence of various factors, among which we can distinguish at least three groups: predisposing, enabling and reinforcing change in health behaviors [4].

Nowadays, examination of health behaviour is regarded as an important method for measuring the health condition of the population, also constituting the foundation of planning and evaluating for health education, developing preventive programs and health promotion projects.

According to the World Health Organization (WHO), health does not only mean the absence of illness or disability, but a state of physical, mental and social well-being [3]. Therefore, assessment of health (in addition to biometric indicators) takes into account the subjective sense of health based on personal experiences and reflection, on what we feel and on what kind of people we are. The sources of emotions are located in us and in our environment, and ‘*a subjective attitude towards own life in the form of its assessment is a consequence of the situations that arose and, simultaneously, constitutes a psychological aspect of the quality of life*’ [5].

As a result, it is worth analyzing and examining whether Canadian Polonia is satisfied with living in this country and how health factors are evaluated, both in terms of subjective assessment (quality of life) and objective assessment (standard of living).

Statistical data confirms that, despite relatively restrictive regulations and plans for their additional strengthening, Canada still remains an attractive country for newcomers and those striving to become newcomers. It receives around a quarter of a million new immigrants every year. Besides Australia, this country has the highest immigration rate per capita in the world. This positive immigration ratio means that Canada has the highest population growth among G8 countries and one of the highest among highly developed countries [6].

Canada is a country with an immigrant origin, which, due to the standard of living-offered, attracts crowds of immigrants from all over the world. Toronto is a modern multicultural metropolis that encompasses the whole spectrum of social and cultural diversity [7]. This is a space with a visible ethnic and racial diversity of inhabitants, which respects the symbols assigned to them. Multiculturalism, defined by Fish [8] as strong, manifests itself, above all, in the ethnic diversity of the inhabitants of such modern cities.

The metropolis best illustrating this state of affairs is undoubtedly the largest city of Canada, Toronto, founded in 1793, which can be called a multicultural capital of the world. Today, it has representatives of over 200 ethnic groups, which makes the city more ethnically diverse than Miami, Los Angeles or New York. According to the latest national census [9], there are 2.5 million inhabitants in Toronto and 5.5 million in the GTA (Greater Toronto Area). In the years from 2001 to 2006, 267,855 immigrants settled within the territory of Toronto [10]. Data from 2011 confirms that 47% of the Toronto population is included in its visible minorities. Reczyńska (2011) presents the most numerous national minorities in the census of 2011 (Table 1). 

Considering the multiculturalism of the GTA and the interpenetration of various cultures, nationalities and ancestral descent, it is important to examine how the community of Polonia assesses its life in this metropolis. Therefore, the main purpose of this research was to determine the standard and quality of life of the Polish community living in the Greater Toronto Area, which includes the city of Toronto and four regions: Halton, Peel, York and Durham. After conducting extensive field studies within this scope, this article will present assessment of both objective factors (standard of living) and subjective ones (quality of life). The respondents’ assessment concerning, among other issues, access to the healthcare system and access to sports facilities in the city will be discussed. Evaluation provided by respondents regarding satisfaction with their general health, mental well-being and the possibility of practicing sports, which is associated with maintaining a healthy lifestyle, will be shown.

The origin of the concept of the quality of life is not a simple, clear and obvious one. The main reason for this state of affairs is its ambiguity, the evolution it has been subject to and the fact that, within various scientific disciplines, numerous synonyms were and are used interchangeably with it. The issue of quality of life has developed differently in psychology, where the of interest was limited to issues related to this area of science, and in medicine. However, this does not mean that they each discipling took advantage of its own achievements, rather they enriched each other by developing new research methods [12].

The issue of living conditions and the quality of life of a population is extremely diverse and requires various socio-statistical analyses. Numerous studies in this area have been undertaken as part of the Integrated System of Research on Households that has been implemented by the Central Statistical Office of Poland since 1982. This research was and is still being conducted by various scientific and research institutions (e.g., Eurostat, CBOS (Public Opinion Research Center)). Until recently, this issue has also been addressed as part of so-called central and inter-ministerial research concerned mainly with social indicators, the concept of families’ standard and quality of life, minimum subsistence, the role of social benefits, social poverty, participation of the population in culture, and transformations in the broadly understood level and structure of consumption.

Indicators of quality of life include the ability to play current life roles, adaptability, psychological well-being and functioning within social groups.

The term ‘quality of life’ appeared in the second half of the 20th century. Research on quality of life was initiated by Allardt, a Finnish scientist, who suggested that research on the standard of living should be extended by two new elements that make up quality of life: an analysis of emotional states (to love) and the sense of existence, of being someone (to be). The beginning of interest in quality of life was caused by disappointment with economic growth and the conviction that increase in material goods was not sufficient to make human life better. Attention was drawn to the fact that, apart from positive effects, economic growth also brings negative ones such as degradation of the natural environment, disintegration of interpersonal relationships and social pathologies. It has been noticed that the satisfaction that a person derives from life does not only depend on the material goods he or she possesses, but also on the ability to satisfy higher needs, such as state of mind, a sense of security, self-fulfillment, and participation in the environment in which we live as residents [13,14].

The scope of the concept of quality of life, [15] suggests that we should distinguish a wide and a narrow approach. Taking into account the nature of the indicators and the level of analysis, the author postulates recognition of quality of life in an objective and subjective sense, and of its relationship with the axiological system. She also puts forward two positions related to different interpretations of needs and value systems. The first, formed on the basis of Maslow’s theory of needs, focuses on the analysis of issues related to the category of scarcity. The second, pursuant to prognostic assumptions based on specific concepts of man and of human personality, emphasizes the development needs of both the individual and society.

Quality of life constitutes an object of interest for economists, statisticians, sociologists and politicians. Human life is of the highest value and its quality determines satisfaction and possibilities of development for each person and for the whole society. The quality of life of the population is associated with socio-economic development and economic growth. It influences one of the factors of economic growth, i.e., human capital. It determines physical fitness, shaping performance, pace of work and forming the psycho-intellectual condition of people through the conditions it creates for development, education and levels of vocational preparation, which develops their creativity and innovation. Furthermore, subjective feelings in society regarding quality of life and prospects for its improvement shape the economic climate, in particular the investment climate, which translates into the economic situation.

There are two approaches to quality of life in the literature on the subject:

- an objective approach, according to which the objective living conditions of people, measured by means of objective variables, such as GDP dynamics, environmental pollution, infant mortality rate, health, and the material and social living conditions of people constitute quality of life;
- a subjective approach that identifies quality of life with the satisfaction of people in various spheres of their lives. According to this approach, quality of life constitutes the individual’s sensation of well-being, his or her satisfaction or dissatisfaction with life.

Similarly, the definition of the World Health Organization includes an objective and subjective aspect since it regards the quality of life as

*‘the manner individuals perceive their life position in the cultural context and system of values in which they live, and in relation to their tasks, expectations and standards set by environmental conditions. Therefore, the quality of life is the same as a comprehensive way of assessing the physical health, emotional state, independence in life and the degree of independence from the environment as well as personal beliefs and beliefs by an individual’.*

[16]

Skrzypek [17,18] defines quality of life as a combination of the objective and subjective elements. The fundamental determinants of quality of life include:

- objective conditions, such as economic conditions, leisure time, social security, housing conditions, the natural environment, health, social environment, etc.;
- subjective conditions, which are perceived in a unique way by each human being, and are manifested in his or her well-being, where assessment of living conditions is expressed in terms of satisfaction, happiness, fears, hope and loneliness.

M. Rapley [19], one of the researchers on quality of life, stated that quality of life is a synonym of happiness, satisfaction with life, freedom from coercion, a state of complete sense of well-being (physical, mental and social) and not only the absence of illness, objectively good functioning, mental balance, prosperity, self-fulfillment, mental comfort, low unemployment, high income, a good life, joy, good life experiences, a life full of meaningful existence.

As Jankiewicz-Siwek and Bartosińska [20] note, there is currently no one universal measure that can be used to assess quality of life. It is described by the application of numerous variables that are expressed in various units of measure. These are qualitative features whose measurement is subjective.

According to T. Słaby [21], quality of life includes all the elements of human life that are associated with the fact of human existence, of being someone and of feeling various emotional states, resulting from, e.g., having a family, colleagues or friends.

From the point of view of social policy, quality of life appears as a synthetic assessment of standard of living (the state of meeting material and non-material needs) and an assessment of the principles of the organization of social life and the nature of social bonds [22];

a measure of subjective satisfaction with life, a measure of well-being, good or bad individual and collective well-being.

The presented definitions of quality of life are closely related to Maslow’s theory of needs, based on the assumption of the hierarchical nature of human needs. The lowest level in the hierarchy is created by physiological needs. The needs for security and safety constitute the next level. Maslow describes physiological needs along with the needs of security and safety as material needs. Social needs constitute the next level in this hierarchy. Among them, the need to belong is fundamental. In the group of social needs, the most important is the need for a sense of human value. Maslow defined material and social needs as deficiency needs. The group of needs related to self-actualization occupies the highest position in the hierarchy. These include the needs for love, truth, justice, perfection, beauty and meaning in life.

The term ‘quality of life’ covers all these presented groups of needs. In addition to material needs, it also takes into account social and spiritual needs. Therefore, quality of life is satisfaction with meeting all human needs.

Słaby [23] believes that *‘standard of living’* should be determined on the basis of a group of objective measures (regarding quantities and values), while quality of life should be assessed mainly with the application of subjective measures (indicators). The former record the existing actual state of affairs, e.g., the level and structure of income, expenditure, consumption, whereas the latter inform about the manner in which this state is perceived and assessed by particular persons or social groups. These are the indicators that are suitable for assessing the quality of life, understood as the level of human satisfaction with the degree of meeting various human needs.

Generally speaking, subjective measures can be divided into those that express value judgments and those that relate to the respondent’s intentions. Value judgments are, in particular, characteristics of the degree of satisfaction (e.g., with work, home, financial situation, etc.) and self-esteem (e.g., whether the person possesses sufficient preparation for the performed job, self-assessment of level of income habitation, standard of provided services, etc.) [24,25].

An important and often ignored procedure is the weighing of these spheres of life by including level of satisfaction, e.g., from family life, or from the use of a professional weight index (a hierarchy of values) in the assessment of these areas of life and activities. Filipp and Ferring [26] confirmed that such a procedure significantly changes the results of the research on the sense of quality of life. In research concerning the quality of life in situations of critical events and illness, the sense of intensification of change as a result of confrontation with the event, the valency of the ‘in plus’ or ‘in minus’ change, as well as the scope, i.e., the number of areas of life in which changes have occurred, are taken into account, along with whether the extent this change is desirable or not.

As Wnuk et al. [27] state, in research on quality of life, a value-based understanding of quality (higher quality, lower quality) is applied more frequently than a descriptive interpretation (different quality, different qualities). Two basic trends in the research and discussion can be distinguished in relation to the possible ways of conceptualizing and operationalizing the category of quality of life. The first one differentiates quality of life in the subjective and objective sense, as well as phenomenalistic and normative understanding. The second results from differences in value systems, ethical attitudes and implemented development concepts [28].

The concept of ‘quality of life conditioned by health’ appeared in medicine thanks to Schipper. In the Polish literature on the subject, it functions in three variants as: 1. health-dependent quality of life, 2. health-related quality of life, and 3. quality of life conditioned by health status. Each manner of conceptualizing quality of life conditioned by health status should relate to three areas: feelings, functioning and the future. The area of feelings concerns the subjective sense of well-being of the individual in all spheres of life, the area of functioning is related to the physical, cognitive and interpersonal activity of the subject, whereas the area of the future is associated with forecasting changes that may occur in the other two areas [29].

Research on quality of life with reference to health condition has been initiated in the field of medicine in order to assess functioning of the patient depending on ailments and limitations resulting from disease [30].

As emphasized by Sęk [31], quality of life, recognized objectively, constitutes a set of human living conditions, objective attributes of the world of nature, objects and culture, as well as objectively assessed human attributes related to standard of living and social position. These objective indicators of quality of life also include the structure of the human body and the level of functioning of the human biological systems, i.e., health. Subjectively, quality of life is defined as a result of the internal processes of evaluation of various spheres of life and of life as a whole. This is the cognitive-experiential (emotional) category, and the subject is the most important, if not the only, source of the data pursuant to which a level of quality of life can be inferred.

As Sęk et al. [32] state, contradiction is expressed in the fact that individuals asked about the source of their quality of life put health first; they also think that a sense of good health, apart from the sense of security and freedom, can be attributed to a happy person and is the most characteristic for the comparison between quality of life and health. However, the real strength of the relationship between satisfaction with life, i.e., the sense of the quality of life, and satisfaction with life is considerably weaker. The contribution of the quality of a healthy life to a global sense of quality of life is significantly smaller than satisfaction with family life, financial and housing conditions, friends, etc.

In the subject literature within the scope of mental health, we find approaches that identify mental health with mental well-being, which has also been reflected in the definition of the World Federation for Mental Health. Within this approach, health is identified with quality of life as one of the dimensions of happiness. However, this is not an attitude that plays a significant role in contemporary health models. With the development of behavioral medicine and health psychology, it has been stated that health can be presented within other theoretical perspectives, biomedical as well as holistic-functional [32].

Quality of life in the city is influenced by a variety of factors of differentiated importance; these include, among others: health, safety, access to services (including public services), the possibility of rest and recreation, the quality and friendliness of space (in particular public spaces, streets, yards, squares), cost of living, transport efficiency, the presence of pollution (especially air and noise pollution), and functioning within the community [33,34].

■ Measurement of quality of life requires designation of the domains (areas) of the quality of life to be observed. The report of the Task Force on Multidimensional Measurement of Quality of Life suggests that measurement of the quality of life within the European Statistical System should cover nine domains (areas). Eight dimensions relate to areas connected with living conditions and the ninth concerns mental well-being: 1. Material living conditions 2. Economic activity and working conditions 3. Health 4. Education 5. Leisure time and social relations 6. Economic and physical security 7. State and fundamental rights 8. Quality of the environment in the place of residence 9. Subjective well-being [35].

The report of the Expert Group on Quality of Life of the European Commission [36] presents a proposal for a system of indicators to measure the quality of life. In the process of its creation, it was assumed that this measurement should be based on both indicators of an objective type (measuring the quality of life of individuals regardless of their personal value) and subjective indicators (constituting the assessment of the quality of life by individuals themselves). Furthermore, a group of 17 basic indicators has been distinguished within the system of indicators, including health.

Despite the extensive literature on the quality and standard of living, there has not been a scientific publication regarding the analysis of the assessment of the level and quality of life of the Polish community living in the Greater Toronto Area before now. This is an important issue from the sociological, geographical, migration and cultural point of view. Health aspects, including an evaluation of satisfaction with the standard and quality of health in the opinion of the Canadian Polonia community, has been undertaken in this research.

## 2. Materials and Methods

### 2.1. The Course of the Research and the Time and Spatial Scope of the Empirical Research

According to Reczyńska [37], the community with the largest number of people of Polish origin in Canada is the province of Ontario. There are almost 161,000 people who declare only Polish origin (62.2% of the whole group) and over 224,000 people who confess to Polish roots. The number of people of Polish origin living in other provinces is much smaller. Comparison of the statistical data clearly shows that the Polish group is concentrated in large cities in Canada and this process is deepening. While in 1971 almost 40% of Canadians of Polish origin lived in the seven largest Canadian cities, in 2001 the same cities were inhabited by 74.5% of those confirming only Polish origin and 44.1% of people with Polish roots.

The capital of Canadian Polonia is Toronto. The city has more than 83,000 people (32% of the whole Polish group) reporting only Polish origin and the same number of people providing some Polish origin. Since the 1940s, Toronto has attracted and continues to attract the largest number of Polish immigrants. Since the 1980s, the Polish community has been mainly concentrated in Mississauga and Brampton, and that is why the research has not only been conducted in the city of Toronto; instead, its coverage included the entire Greater Toronto Area in which the population of Polish origin lives.

There are familiar publications by Canadianists who raise in their studies aspects of history, multiculturalism, migration, Polish pastoral activity, causes of emigration and Canada’s policy towards people who have e obtained immigrant status However, research taking into account the quality and standard of life of the Polish community in Canada in the context of health would fill a gap in research on the Polish diaspora in Canada. This research complements the historical threads concerning emigration from Poland, in the context of the quality of life of the current Polish community living in the Greater Toronto Area. A large wave of Poles emigrating to Canada took place in the 1960s and 1970s. Today, the people who arrived at that time are 60 years old or older. They are often people currently retired, often requiring medical care, and health has a significant impact on their assessment of the quality and standard of living.

In 2011, people aged 60 and over constituted 11% of the world’s population; however, by 2050, already one in five people will be in this group. 31.8% of the Canadian populations will be older than the age of 60 [38]. The possibility of active and effective mobility in older people is an important element influencing their quality of life, particularly as it determines their accessibility to health care, cultural institutions, trade and services [39].

According to Reczynska, [40], in 1991 Poland was in second place (after Hong Kong) in the summary of ten countries constituting the most important sources of immigration to Canada. In 1996, Poland shifted to sixth place (after Hong Kong, China, India, the Philippines and Sri Lanka); however, it was still ahead of other European countries. The last population census including persons declaring Polish origin is available from 2016. At that time, 259,715 people declared Polish origin in the area covered by the survey (GTA), which is a 4% share of the Polish community in the total of all GTA residents (6,417,516). Such a large number of the Polish diaspora in the GTA, clearly demonstrating its distinctiveness in multicultural Canada, constitutes an important topic for exploring and gaining information for sociological research concerning this population in Canada. Taking into account the fact that such a large group lives not only in the city of Toronto but within the entire GTA area, the research was also conducted outside Toronto itself.

Contemporary distribution of Poles in Canada has been conditioned, on the one hand, by the processes of development of the young state and, on the other hand, by social changes in the Polish community itself. Until the 1930s, immigrants from Central Europe were directed to agricultural areas in the provinces of Manitoba, Saskatchewan and Alberta by the Canadian authorities. It was only after the Second World War that Poles began to settle mainly in cities. Urbanisation processes resulted in the fact that, as early as the 1970s, 80% of Poles lived in cities, mainly in the large agglomerations of the provinces of Ontario and Quebec. Nowadays, cities, particularly Toronto and subsequently Montreal, Ottawa, Edmonton, Hamilton, Vancouver, Winnipeg, and Calgary, constitute the main centres of the Polish community. Representatives of the most recent waves of Polish migrants from the second half of the 20th century settled mainly in Toronto, hence the strong numerical dominance of the Polish community living there. The most active organizations operate in this city and most of the Polish press titles are published here [41]. 35,151,728 people lived in Canada in 2016, and 1,106,585 inhabitants were of Polish origin, 3.9% of the total population. Ontario, where the Greater Toronto Area is located, is the province with the greatest number of inhabitants of Polonia. 523,490 such people lived in Ontario in the year 2016, while the largest number of people of Polish descent in relation to all residents is in Manitoba, where the percentage of Poles is as high as 6.9%.

Most Polish people live in Mississauga, in the county of Peel. This population equals 43,350 people, which is 6% of the total population in Mississauga. A significant number of people of Polish origin live in the county of York, in Vaughan. This number includes 18,265 people, which is 5.96% of the total population of the city. The smallest number of Polish people lives in two towns in the county of Durham, the city of Brock, with 335 people of Polish descent, and the city of Scugog, with 590 people. In 2016, Statistics Canada published census data which confirms that 34 million Canadians use over 200 languages on a daily basis. In the table of Statistics Canada (2016), Polish is the eleventh most widely spoken language in Canada.

The Greater Toronto Area includes the regions of Peel, Halton, York, Durham and the city of Toronto itself. The time period of the conducted research covered the years 2017, 2018 and 2019. In 2017, pilot studies were conducted, whereas the actual research in Canada was carried out in two stages. The first stage of field studies took place during the period from 16 August 2018 to 18 September 2018, and the second stage from 10 September 2019 to 19 September 2019.

One of the quantitative studies uses a diagnostic survey method in the form of a questionnaire. This method has been applied in this research. The results of the research conducted among the Polish community living in the Greater Toronto Area constituted the fundamental empirical base. The empirical part of the research took the form of a diagnostic survey, conducted by the application of a representative method among the population of Polonia of the Greater Toronto Area. The appropriate research covered a total of 612 respondents, including 583 questionnaires used for the analysis. The research was carried out among individuals who were at least 15 years old.

In order to make the research as representative as possible, seven age groups have been differentiated, with the use of a pre-conceived division. These were the following age groups: 15–19, 20–24, 25–29, 30–39, 40–49, 50–59, and over 60 (women and men were additionally included separately). Conducting research among people aged 15 and over allowed certainty that respondents understood the questions and that the answers provided were reliable. In this article the research results are presented for all age groups together.

The questions applied in the questionnaire were formulated in such a way as to refer to the experiences of the respondents in 2017 and 2019, as well as from the period of the last several years. This approach has enabled the capture of an average assessment of the quality and standard of living of the surveyed group of respondents. During the analysis of the quality and standard of living, factors such as gender, age, education, religion, profession, achieved material level, size of household, and regulated residence status were used. This has allowed for a more precise description of the examined group and identification of the relationships between interesting aspects.

The survey questionnaire was prepared in two languages: Polish and English, and consisted of 17 questions plus 15 questions in the demographic section. In the quantitative research, answers are most often ‘provided’ and respondents indicate one of them.

The survey questionnaire included questions concerning the following issues:

■ Assessment of specific elements of the standard of living in Toronto
■ Assessment of specific elements of standard of living associated with Polish origin in Toronto
■ Assessment of the possibility of meeting the person’s needs in the city, within the scope of particular elements
■ Assessment of satisfaction from particular areas of life
■ Social, demographic and economic features of the respondents

In the conducted study, one of the questions concerned the sense of Polish national identity. Respondents were asked if they feel Polish. 97.4% of the respondents answered ‘yes’. Only 0.6% of the respondents answered that they did not, and 2% did not have an opinion on the subject.

### 2.2. Model of Objective Factors (Standard of Living) and Subjective Factors (Quality of Life)

One of the main aims of the research was to construct an individual model of objective factors (standard of living) and subjective factors (quality of life) and to examine whether the suggested indicators/measures interact with each other. The model has been created on the basis of the prepared questions in the survey questionnaire, taking into account the specific factors that have and may have an impact on the overall assessment of the standard and quality of life. The described model including health factors is presented in Table 2. In the demonstrated research, individual relationships will not be described, and only the factors examined during the research in the Greater Toronto Area will be listed.

Terms such as ‘living conditions’, ‘standard of living’ or ‘living standards’ are used interchangeably with the term ‘quality of life’. Some use these terms as synonyms. It should be noted that ‘standard of living’ is determined by objective living conditions, and ‘quality of life’ is also influenced by subjective factors, such as aspirations and the degree of their satisfaction or perception. The model of objective factors (standard of living) and subjective factors (quality of life) put forward has included demographic factors, social factors, cultural factors, economic factors, political and legal factors, educational factors, geographical factors, economic and health factors that attention has been devoted to in this study.

This is a novel research tool to assess the quality and standard of life of the population. This model was described in the article entitled ‘*Evaluation of the selected components of standard of living and quality of life within a Polish diaspora: the example of the Greater Toronto Area*’ [42] and particular factors will gradually be described in later scientific studies. This article pertains to the health factors influencing the level and quality of life of the Polish community in the GTA.

Assessment of personal well-being, mental health and health constitutes a significant aspect when describing the quality and standard of life. Therefore, the research has included health factors to which due attention has been paid. Table 2 presents the model of objective health factors (standard of living) and subjective health factors (quality of life) that have been considered during the empirical research.

In the suggested model of the quality and standard of living, each indicator has been matched with particular indicators/measures, in the factors related to the assessment of the both the level and the quality of life. Mutual correlation between the selected subjective and objective factors constitutes the main assumption of the research.

Respondents were asked about access to the healthcare system, leisure time, conditions for rest and relaxation, access to tourism, possibility of practicing sports (standard of living) and about their assessment of satisfaction with health, mental well-being and the possibility of practicing sports (quality of life).

## 3. Results

In the survey questionnaire, subjects from Canadian Polonia were asked about assessment of subjective and objective aspects related to health. Answers to the individual questions can be found in Table 3 (objective conditions, assessment of living standards) and Table 4 (subjective conditions, assessment of the quality of life). The respondents were asked to rate each of the listed elements, marking their opinion on a six-point Likert scale-from *I am very satisfied* to *I am very dissatisfied*; *I have no opinion* (in the case of questions regarding assessment of standard of living); and to assess the quality of life (subjective factors) where they rated their assessment of health factors from *very good* to *very bad* and *I have no opinion*. The six-point Likert scale has also been used in the question about problems related to health elements, and which, according to the respondents, should be addressed first. The respondents have rated the scale of the problem from *a very important problem* to *an unimportant problem at all*, *I have no opinion*. Table 3 presents the answers of the respondents to the questions concerning assessment of standard of living in the context of the health factors.

Respondents were asked to assess accessibility to the healthcare system, possibilities related to leisure time, accessibility to rest, relaxation and tourism, as well as access to practicing sports (sports facilities: sports fields, tennis courts, swimming pools). In this question, from all the provided answers, the majority of the respondents answered that they are satisfied with possibilities for leisure time (361 answers). The largest number of ‘I am very satisfied’ answers referred to access to the healthcare system: 123 answers along with 260 ‘*I am satisfied*’ answers. With such a high satisfactory response concerning access to the healthcare system, at the same time the largest number of respondents replied that they were dissatisfied with this aspect. There were 73 such answers. 94 of all respondents have replied that they have no opinion regarding accessibility to rest, relaxation and tourism.

One of the research objectives was to check how people of Polonia in the Greater Toronto Area assess the quality of life (subjective assessment) of the selected health factors. Table 4 presents the answers to this question.

As in the question regarding standard of living, the aspect related to satisfaction with one’s own health was very highly rated by respondents. As many as 120 people evaluated their health satisfaction as very good and 263 as good. Only 16 out of 583 respondents answered that their health has been assessed as very bad and 32 as bad. From the provided answers, it can be concluded that the surveyed people from Polonia in the Greater Toronto Area are also satisfied with their mental well-being, as 114 people rated their mental state as very good and 239 as good. Almost a quarter of the respondents (150 people) answered that they are quite satisfied with the possibility of practicing sports (this was the most frequently provided answer among all answers in the category ‘*quite satisfied*’). The majority of the given answers also indicate satisfaction with the manner of spending their leisure time, assessing it as good, with 361 respondents providing such an answer.

In the prepared questionnaire, subjects were also asked which issues should be addressed in the first place to improve their overall level and quality of life. Two aspects related to health have been included in these suggested aspects. These were improvement of access to the healthcare system and increase in sports infrastructure (development of sports facilities). The answers to this question are in Table 5.

According to the respondents, improvement in access to the healthcare system constitutes a very important problem and should be addressed first. There were as many as 231 and 152 replies from the respondents’ opinions that this is an important problem. Only 5 respondents believe that improving access to the healthcare system is not a significant problem and 27 subjects think that it is a minor issue. After receiving such answers, it can be safely concluded that improvement of the quality of life related to access to the healthcare system constitutes a very important and substantial issue for people of Polish origin living in the Greater Toronto Area.

The respondents assessed issues related to the development of sports infrastructure as less important. 94 people have no opinion at all and only 115 think that this is a very important problem that should be addressed immediately.

Another question concerning issues related to health in the survey questionnaire was: ‘In the last twelve months did it happen to you or anybody in your household that there was no money for expenses related to treatment, or purchase of medicines?’ The respondents had a choice of three answers: ‘No, there was not such a situation’; ‘Yes, there was such a situation’; ‘I do not know, I do not remember (I do not want to answer)’ and 498 people have replied that there was not such a situation. i.e., that they would run out of money for expenses related to treatment or purchase of medicines they needed during the last year. 65 people refused to provide this answer, and 20 respondents admitted that there was a situation where they did not have enough money to cover the costs of treatment last year.

## 4. Discussion

Value judgement obviously depends on the structure of needs and the individual system of values, and, in particular, on the individual’s concept of the meaning of life. Even a fragmentary review of the literature on quality of life, its nature, objective and subjective conditions [43,44,45], methods of research on the level of quality of life, specific aspects of happiness, especially work, as predictors of quality of life, and the relationship between quality of life and health [46] confirms a considerable diversity of approaches and much contradiction in the results of the empirical research.

Numerous studies, both on large population samples, conducted mainly in the USA [47] as well as on groups of healthy and sick people after severe accidents demonstrate that objective living standards are not clear predictors of the sense of the quality of life. Due to the fact that specific people have a much higher financial level (e.g., as a result of a large win in games of chance), and others have objectively poor health (e.g., as a result of spinal injury) it cannot be predicted that the first group will obtain much higher results in measurements of the sense of satisfaction with life. This applies particularly to measurements of the quality of life projected in the past and into the future.

The research conducted by Brickman and others [48] clearly indicates that, while determining quality of life in people who were participants in critical life events (mental health) during the period of the research, measurements of quality of life related to the time before these events, the time associated with these events and future time display significant differences. Therefore, it should be taken into account that the results will be completely different and assessments of the quality of (a) present, (b) past and (c) future life will perform different functions.

Considering important methodological problems, it should be noted that, even when we assume that in the study of the quality of life, we are guided by the individual, subjective assessment of the subjects, care for minimizing ‘measurement errors’ is of great importance. The research [49] confirms that respondents give higher values for assessment of satisfaction with life in a situation of direct conversation with the researcher than in the case of completing a questionnaire, and that global assessments of quality of life in general are much more influenced by the current situation and the present emotional state of the respondents (mental health, well-being, general health) than when the general measurement of quality of life constitutes the result of the sums of component assessments concerning various distinct areas of life and activity.

While comparing specific measures of quality of life, satisfaction and various forms of optimism with health, the most interesting and important aspects regarding health psychology arise. The effects of satisfaction with life, optimism and various forms of trust in life on the development of health, health protection [50,51,52,53] and effectiveness of coping with disease [54] have been widely examined.

The sense of quality of life dependent on health is a very good indicator of health condition [55], and also influences the subjective perception of the person’s own life situation. The sense of quality of life is shaped by numerous factors, not only related to the characteristics of the person, but also to an understanding of physical health [56], type of disease and the environment in which the patient lives [57]. Family relations, relationships of youth with parents and home life have a significant impact on the sense of the quality of life [58]. Generally speaking, it can be stated that the sense of quality of life dependent on health constitutes a psychological construct describing the physical, mental, social, psychological and functional aspects of well-being and functioning from the point of view of each person [55].

Within the scope of psychology, [59] presents an innovative approach to defining and perceiving quality of life. Here, quality of life can be understood in two ways: either as the sensation of the person’s own life as a result of getting to know it, or as the sensation of the person’s own life by experiencing it. Quality of life can be considered in the dimensions of reflection on the course and the present state of one’s own life, one’s own health, and the experience of various mental (subjective) states during life. Both described solutions refer to the subjective sphere of quality of life, as two aspects of the individual assessment of quality of life. In the event of feeling the person’s own life by getting to know it, reflective awareness constitutes the main tool for assessment of its quality.

It is worth discussing the results of the research on perception of quality of life by Poles in the 21st century in Poland. According to the research carried out by Social Diagnosis, since 2000 an improvement in the assessment of the current lives of Poles can be observed. An increasing percentage of people evaluate their current lives as at least successful. Furthermore, the sense of happiness in society is gradually increasing and the percentage of unhappy people is decreasing [60,61]. The psychological well-being of Poles has been increasing considerably since the beginning of 2000. Assessment of life satisfaction, according to the assumptions of Social Diagnosis, is

■ largely influenced by so-called partial satisfaction, i.e., satisfaction with particular areas of life.
■ In the years 2000–2013, on the basis of the survey conducted by Włodarczyk [62], the greatest increase in satisfaction in Poles concerned: the sense of safety in the place of residence, the manner of spending free time and the person’s own education.
■ The smallest included: satisfaction with sexual life, relations with colleagues and loved ones in the family and satisfaction with marriage.
■ According to the analyses conducted at Social Diagnosis, age has been the most important factor shaping the well-being of Poles for several years. The older the person was, the worse the mental condition of this person was.
■ The second factor in terms of importance for overall mental well-being was marriage, the third was household income and the fourth was related to the number of friends.

Analyzing the data concerning the issues related to satisfaction with the health condition of the individual, in 2000 Poles assessed it at 3.24 on a 6-point-scale, where 1 meant very satisfied and 6 meant very dissatisfied. In 2005, the assessment was 3.09, in 2007 it was 3.13, in 2009 it amounted to 3.0, in 2011 the result was 2.93, and in 2013 it was 2.89, which means that Poles assessed their health condition to be better with each year of the survey as compared to previous years.

Surveys regarding the quality of life and satisfaction of Poles have also been conducted by CBOS, the Public Opinion Research Centre, periodically since the 1990s. The survey conducted in 2019 confirms that almost two fifths of Poles (35%) regard themselves as people who are lucky. The number of those who feel that they have bad luck in life is three times smaller (11%). Every second respondent (54%) answered ‘difficult to determine’ [63].

Similar to the research conducted by Social Diagnosis, CBOS also carries out surveys on partial satisfaction influencing general satisfaction in the life of Poles. The respondents assessed their health condition to be good in the years 2000–2013. This was the opinion of half of the respondents, while on average about a quarter were dissatisfied. Statistical analysis conducted on the basis of the results of the survey carried out by CBOS confirms that future prospects, place of residence, health condition and marriage are most strongly connected with the general sensation of satisfaction.

Among the values respected by Poles (percentage of the respondents who indicated specific values as the most important in their lives) in 2005 (69% of indications), 2010 (74% of indications) and 2013 (74% of indications), health turned out to be an important value and was ranked second after family happiness as the most important value in life. The results of CBOS studies on these respected values are also confirmed by studies conducted in 2011. The survey was carried out among 1000 adult Poles over 15 years of age

■ (representative sample, nationwide). The sample was selected according to statistics of the Central Statistical Office (GUS) by gender and place of residence. The survey was conducted by Pentor Research International in May-June 2011.
■ Among the values that people subscribe to, and the most important ones for the respondents in order of significance (percentage of indications, *N* = 1000), health was second (79.6%), after children and family (79.9%) [64].

Research conducted by Panek and Zwierzchowski [35] 2019) concerned assessment of the quality of life of Poles from a national perspective. The survey adopted the structure suggested by the European Statistical System and concerned nine different aspects. Evaluation of quality of life in terms of health satisfaction constituted one of these. The conducted research confirms that the quality of life is considerably and positively determined by being a pensioner rather than being unemployed, and by being a disability pensioner rather than a professionally inactive person, in the area of health. A significant increase in quality of life in the area of health is fostered by an increase in the level of education, living in smaller urban centers, but not in the rural areas, and being a member of more numerous households. On the other hand, quality of life in the area of health significantly decreases with age and especially for women. The increase in quality of life in the area of health is accompanied by a fall in restrictions on access to medical and dental care due to financial reasons and a fall in restrictions on performing activities for health reasons [65]

There are studies concerning older people in the scientific literature on perceptions of health and quality of life. Moore et al. [66] reported that 11 out of 17 studies obtained a strong positive result between good health and satisfaction with quality of life. Raphael et al. [67] also noted a positive link between quality of life and the health of older people in their studies, indicating that healthy people, without chronic diseases, assess their standard of living to be good.

According to Farquhar [68], living in a desired environment significantly influences the quality of life. Similar findings were made by Stuifbergen et al. [69] as well as Bowling et al. [70]. Sparks et al. [71] concluded that the only significant positive factor that has an impact on life satisfaction is good health and a well-maintained social position.

In contrast, McCamish-Svensson et al. [72] investigated the relationship between support (family and friends) and life satisfaction. They found that satisfaction from contact with siblings was associated with life satisfaction among people over 80, which also significantly contributed to their mental and emotional well-being, which influenced overall good health.

Since 1994, the Quality of Life Research Unit at the University of Toronto has been studying the quality of life of adults with physical or psychiatric disabilities and children with developmental disabilities as well as the elderly, young people and adults in the general population. The studies were conducted among Canadian citizens, in 40 small groups in different locations across the country, to discuss the factors which are important in life and what affects quality of life [73]. Respondents provided answers in various thematic areas: democratic rights, health care, education, environment, social programmes and conditions, community, material conditions, economy and employment, and government policy. The studies confirmed that the state plays a fundamental and integral role in providing the components of a good life and influences the overall assessment of quality of life by citizens, especially in the aspects of health and education.

Paskulin from the Federal University of São Paulo in Brazil and Molzahn [74] from the University of Victoria in Canada have published data on the quality of life of older people. The studies were carried out on a random 202 older people from Canada and 288 respondents from Brazil. The authors wanted to examine whether there are differences in the quality of life of people in selected regions of Brazil and Canada and whether health satisfaction, aspects concerning the meaning of life, leisure opportunities, satisfaction with housing environment, satisfaction with personal relationships, satisfaction with the ability to perform life activities and possession of adequate financial resources contribute to the perception of the quality of life of older people in the selected regions. As the results of the studies confirmed, evaluation of overall quality of life as well as physical, psychological and environmental areas were higher in the Canadian sample. The authors stressed that health satisfaction, sufficient amounts of money and leisure opportunities contributed to the assessment of a high quality of life in both countries. Health satisfaction was the strongest contributor to a high quality of life, both in Canada and Brazil.

Chappell [75] compared the quality of life of older people living in mainland China and those living in Canada. Despite considerably larger levels of poverty, worse education and a health status that was perceived as lower in China, she found that, despite this, an important factor in the quality of life in both cultures is social support and one’s own health.

Other studies on the quality of life in the Chinese diaspora were conducted by Da and Garcia [76] within the scope of socio-cultural adaptation and change in quality of life in the settlement of recent older Chinese immigrants in Canada. The research was carried out among 31 older women and men who recently emigrated from China to Canada, with the main aim of examining the perception of quality of life in the new place. Their overall perception of quality of life after immigration was characterised by losses and gains as well as influenced by multifaceted factors, such as language, intergenerational relations, economic status and those supporting them.

In the literature, there are also studies on the quality of life of Canadian indigenous peoples, known as Aborigines, that include three groups of Aboriginal peoples in accordance with the Canadian constitution: Indians, Inuit and Métis. Familiar studies include that by Salee entitled ‘Quality of life of Aboriginal people in Canada. An Analysis of Current Research’ from 2006 and studies by Kant et al. [77] who published data on the quality of life of Canadian indigenous people in 2013. The research team conducted these surveys in the province of Ontario (population 600, 120 households) and in British Columbia (population 1500, 275 households). During the survey, 316 questionnaires were collected. The questionnaire included questions on satisfaction with general welfare, wellbeing, education, employment, health, housing, income, and land use. In order to assess the level of satisfaction, the respondents used a 7-point Likert scale (1 = extremely dissatisfied and 7 = very satisfied). The studies confirmed that the Aboriginal population is not satisfied with their health condition; there are often mental and psychological problems among this population, in both women and men. The research shows that development of national politics based on drawing attention to mental health in Aborigines and assistance from the government in this regard would significantly improve the quality of life of these peoples in Canada, around 1.7 million people, representing 3.8% of the Canadian population.

Studies regarding quality of life in different cultures were also conducted. The LEIPAD project [78] confirmed that good health has a significant impact on the quality of life of older people in Finland, Italy and the Netherlands.

The World Health Organization Quality of Life Instrument—Older Adults Module (WHOQOL-OLD) aimed to develop a programme for intercultural measurement of the quality of life of the population. The instrument was tested in 22 countries [79]. While conducting the studies it was found in eight countries that the impact of chronic diseases had a visible effect on the negative assessment of quality of life regardless of cultural differences between countries [80].

Tesch-Romer et al. [81] conducted studies in Norway, Germany, Spain and Israel, hypothesising that good health and quality of life depend largely on good relations within the family, but also on the type of welfare state. In three out of four countries, the results confirmed the assumption that the relationship between family support and quality of life is high where there are low transfers to the welfare state.

Keith et al. [82] conducted studies in Botswana, Ireland, the United States and Hong Kong. They did not directly assess quality of life; however, they described the available health services, morbidity, and mortality as well as physical and functional status in each culture. After the conducted studies, they concluded that in order to understand the influence of health on the quality of life and functioning of people, the analysis must be tailored to the surrounding living conditions that affect overall life satisfaction. They noticed in Botswana and Hong Kong that poor quality of life is often due to a lack of basic resources, while in Ireland and the United States, they did not notice this kind of dependence.

Minicuts et al. [83] developed a mutual database to identify the common factors of quality of life in Finland, Italy, the Netherlands, Spain, Sweden and Israel. The database included sociodemographic variables, health habits, health condition, physical functioning, social relationships and support as well as health and social services. In each country, respondents indicated that health factors and good health are very important. The Finns and the Swedes rated their quality of life the highest whereas Spaniards regarded their quality of life as the lowest.

## 5. Conclusions

Considering the large number of people of Polish origin living in the described area, an important topic related to the assessment of the level and quality of life as well as health factors by Polonia living in Toronto has been discussed. Such extensive research in this field has not been conducted before. The presented material constitutes only part of the research and the issues that have been raised and examined during field studies among the Polish community in the Greater Toronto Area in the years 2017–2019. Based on the provided data concerning health issues, it can be summarized that:

▶ Canadian Polonia is most satisfied with possibilities for leisure time and accessibility to rest, relaxation and tourism,
▶ Canadian Polonia highly evaluates satisfaction with general health and general mental well-being
▶ Canadian Polonia believes that improvement in access to the healthcare system constitutes a very important aspect that should be addressed by city authorities
▶ A considerable proportion of the respondents declare that over 12 months they have not run out of money for expenses related to treatment or purchase of essential medicines.

While characterizing the level and quality of life, a general model of quality of life, even assuming an approximate one defining the framework for further analysis and empirical research, would be acceptable. Such a model would organize the elements comprising assessment of quality of life to a level corresponding to individual variables or their groups, as well as determine the manner of their inclusion. However, there is no model that is generally accepted in the literature. As a result, the author of the text has put forward her own model, taking into account both elements belonging to objective conditions (standard of living) and subjective ones (quality of life).

The objective of the research conducted among the Polish community in the GTA was to construct a model on the basis of which it was possible to examine the Polish community with regard to numerous areas concerning the level and quality of life (in the following aspects: demographic, social, cultural, economic, legal, educational, geographical, health and economic). The model of indicators regarding the level and quality of life in relation to health aspects presented in Table 2 may provide a ready-made scheme to explore similar relationships in another research group in a different part of the world.

## Figures and Tables

**Table 1:** The most numerous national minorities in the census of 2011.

Immigrants in Toronto	Population (Percentage of the Population)
South Asia	298,372 (12%)
Chinese people	283,075 (11.4%)
Black Canadians	208,555 (8.4%)
Filipino	102,555 (4.1%)
Latin America	64,860 (2.6%)
West Asia	42,755 (1.7%)
Southeast Asia	37,495 (1.5%)
Koreans	34,220 (1.4%)
Arabs	22,485 (0.9%)
Japanese people	11,965 (0.5%)
immigrants of British descent (England, Scotland, Ireland)	19%

Source: Author’s own compilation based on [11].

**Table 2:** Model of health factors; objective (quality of life) and subjective (quality of life).

Objective Conditions	Indicators/Measures Standard of Living	Subjective Conditions	Indicators/Measures Quality of Life
**Health factors**	- access to healthcare system - possibility of spending leisure time - accessibility to rest, relaxation and tourism - access to practicing sports	**Health factors**	- satisfaction with health - satisfaction with mental well-being - satisfaction with possibility of practicing sports

Source: Author’s own compilation.

**Table 3:** Assessment of the standard of living of the selected health factors by Polonia in the Greater Toronto Area.

Satisfaction with Areas of Life	Very Satisfied	Satisfied	Quite Satisfied	Dissatisfied	Very Dissatisfied	I Have no Opinion
Access to healthcare system	123 (21%)	260 (45%)	100 (17%)	73 (12.5%)	11 (2%)	16 (2.5%)
Possibility of spending leisure time	40 (7%)	361 (62%)	38 (6.5%)	60 (10%)	22 (4%)	62 (10.5%)
Accessibility to rest, relaxation and tourism	115 (20%)	268 (46%)	63 (11%)	26 (4%)	17 (3%)	94 (16%)
Access to practising sports (sports facilities)	109 (19%)	209 (36%)	156 (27%)	42 (7%)	17 (3%)	50 (8%)

Source: Author’s own compilation based on the conducted research.

**Table 4:** Assessment of the quality of life of the selected health factors by people of Polonia in the Greater Toronto Area.

Element of Meeting Needs in the City	Very Good	Good	Quite Good	Bad	Very Bad	I Have no Opinion
satisfaction with health	120 (21%)	263 (45%)	96 (16%)	32 (5%)	16 (3%)	56 (10%)
satisfaction with mental well-being	114 (20%)	239 (41%)	98 (17%)	12 (2%)	17 (3%)	103(17%)
satisfaction with possibility of practising sports	107 (18%)	211 (36%)	150(27%)	48 (8%)	19 (3%)	48 (8%)
satisfaction with possibility of spending leisure time	40 (7%)	361 (62%)	38 (6%)	60 (10%)	22 (4%)	62 (11%)

Source: Author’s own compilation based on the conducted research.

**Table 5:** Which of the following problems should be addressed in the first place? Please rate each aspect.

Problems in the Greater Toronto Area	Very Important Problem	Important Problem	Quite an Important Problem	Problem of Minor Importance	Unimportant Problem at all	I Have no Opinion
improved access to healthcare system	231(39.5%)	152 (26%)	145 (25%)	27 (5%)	5 (0.5%)	23 (4%)
increase in sports and recreation infrastructure (development of sports facilities, e.g., construction of swimming pools, tennis courts)	115 (20%)	193 (33%)	138 (23.5%)	26 (4,5%)	17 (3%)	94 (16%)

Source: Author’s own compilation based on the conducted research.

## Data Availability

Anonymous questionnaires completed by respondents are stored at the author’s home.
